# Supplementary material for: Adverse pregnancy outcomes in women with diabetes-related microvascular disease and risks of disease progression in pregnancy: A systematic review and meta-analysis
Source: PLoS Med. 2021 Nov 22;18(11):e1003856. doi: 10.1371/journal.pmed.1003856 (PMC8654151; doi:10.1371/journal.pmed.1003856)
Supplement: S2 Appendix — (DOCX) [file pmed.1003856.s002.docx]

**S2 Appendix – Search Strategy**

**EMBASE**

1. exp DIABETIC NEPHROPATHY/ OR exp DIABETIC RETINOPATHY/ OR exp DIABETIC NEUROPATHY/
2. diabet*.ti,ab.
3. retinopath*.ti,ab
4. neuropath*.ti,ab
5. nephropath*.ti,ab
6. 3 OR 4 OR 5
7. 2 AND 6
8. 1 OR 7
9. exp PREGNANCY/
10. pregnan*.ti,ab
11. 9 OR 10
12. 8 AND 11
13. 12 [Limit to: Publication Year 1990-2019]

**Medline**

1. exp DIABETIC NEPHROPATHIES/
2. exp DIABETIC RETINOPATHY/
3. exp DIABETIC NEUROPATHIES/
4. diabet*.ti,ab
5. retinopath*.ti,ab
6. neuropath*.ti,ab
7. nephropath*.ti,ab
8. 5 OR 6 OR 7
9. 4 AND 8
10. 1 OR 2 OR 3 OR 9
11. exp PREGNANCY/
12. pregnan*.ti,ab
13. 11 OR 12
14. 10 AND 13
15. 14 [Limit to: Publication Year 1990-2019]

**Cochrane**

1. pregnan*
2. diabet* nephropathy*
3. diabet* retinopathy*
4. diabet* neuropath*
5. MeSH descriptor: [Diabetic Retinopathies] explode all trees
6. MeSH descriptor: [Diabetic Nephropathies] explode all trees
7. MeSH descriptor: [Diabetic Neuropathies] explode all trees
8. MeSH descriptor: [Pregnancy] explode all trees
9. #1 or #8
10. #2 or #3 or #4 or #6 or #7
11. #9 and #10 [publication year 1990-2019]
