# Supplementary material for: Adverse pregnancy outcomes in women with diabetes-related microvascular disease and risks of disease progression in pregnancy: A systematic review and meta-analysis
Source: PLoS Med. 2021 Nov 22;18(11):e1003856. doi: 10.1371/journal.pmed.1003856 (PMC8654151; doi:10.1371/journal.pmed.1003856)
Supplement: S3 Appendix — (DOCX) [file pmed.1003856.s003.docx]

**S3 Appendix – A priori outcomes and risk factors assessed in the systematic review**

**Table 1- A priori outcomes assessed in the systematic review**

| **Maternal obstetric outcomes** | **Fetal/neonatal outcomes** | **Maternal disease outcomes** |
| --- | --- | --- |
| - Miscarriage (pregnancy loss at <24 completed gestational weeks) - Pregnancy-induced hypertension - Pre-eclampsia - Preterm birth (at <37 and <34 completed gestational weeks) - Mode of birth (any vaginal delivery versus any caesarean section). | - Congenital anomalies - Perinatal mortality (stillbirth or neonatal death) - Small for gestational age birthweight - Large for gestational age birthweight - Apgar scores at 5 minutes post birth - Umbilical cord arterial pH at birth - Neonatal hypoglycaemia - Admission to neonatal intensive care. | - New/progressive diabetic retinopathy - Blindness secondary to diabetic retinopathy - New/progressive diabetic nephropathy - End-stage renal disease secondary to diabetic nephropathy - New/progressive neuropathy. |

**Table 2 - Risk factors assessed for association with progression of diabetic vasculopathy in pregnancy**

| **Risk factors** |
| --- |
| - Maternal age - Maternal ethnicity - Time since diagnosis of diabetes (years) - Severity of vascular complication peri-conception - Body mass index (kg/m^2^) - Smoking status |
