# Supplementary material for: Adverse pregnancy outcomes in women with diabetes-related microvascular disease and risks of disease progression in pregnancy: A systematic review and meta-analysis
Source: PLoS Med. 2021 Nov 22;18(11):e1003856. doi: 10.1371/journal.pmed.1003856 (PMC8654151; doi:10.1371/journal.pmed.1003856)
Supplement: S4 Appendix — (DOCX) [file pmed.1003856.s004.docx]

**S4 Appendix: Table of included studies.**

| Study | | | | | | Population | | | Exposure | Outcomes |
| --- | --- | --- | --- | --- | --- | --- | --- | --- | --- | --- |
| Author | **Year** | **Country** | **Data source** | **Data collection** | **Total no. women** | **Type of diabetes** | **Inclusion criteria** | **Exclusion criteria** |  |  |
| Garner, PR[1] | 1990 | Canada | Not stated | Not stated | 334 | Not specified | All pregnant diabetic women who attend the maternal-fetal day unit at the hospital. | None stated | White class D/F/R | Pre-eclampsia, perinatal mortality |
| Schoetzau, A[2] | 1990 | Germany | Not stated | Not stated | 195 | T1DM / T2DM, GDM | Infants of women with gestational, type 1 or type 2 diabetes | None stated | White classification | Neonatal morbidity, preterm <37/40 |
| Hoshi, J[3] | 1991 | Japan | Not stated | Not stated | 160 | IDDM/NIDDM | Infants of diabetic mothers admitted to the hospital | None stated | Retinopathy | Congenital anomaly, LGA, SGA, preterm <37/40 |
| Ayed, S[4] | 1992 | France | Ophthalmic examinations | Prospective | 93 | T1/2 DM | Women with pre-existing diabetes, compared to diabetic non-pregnant women | None stated | Retinopathy | Retinopathy deterioration |
| Rosenn, B[5] | 1992 | USA | Patient reports, retinal screening | Prospective | 154 | T1DM | T1DM with history of ketoacidosis, ≥2 ophthalmic examinations | None stated | Retinopathy, severity of retinopathy | Retinopathy deterioration |
| Combs, CA[6] | 1993 | USA | Not stated | Retrospective | 311 | Not specified | Diabetes mellitus diagnosed pre-pregnancy, completion of at least one 24hour urine collection before 20 weeks of pregnancy, no evidence of urine infection at the time of this collection, creatinine excretion rate of at least 10mg/kg/day in the specimen, pregnancy that passed 20 weeks gestation | Women with gestational diabetes | Nephropathy | Pre-eclampsia, preterm <37/40, preterm <34/40 |
| Chen, HC[7] | 1994 | UK | Retinal photographs, biochemistry | Prospective | 22 | DM | Diabetic pregnant women | None stated | Retinopathy severity | Retinopathy deterioration |
| Chew, EY[8] | 1995 | USA | Patient reports, fundal photographs, biochemistry | Prospective | 140 | IDDM | Pregnant women with IDDM. Enrolled before or within 21 days of conception. | <2 fundal photographs, fetal loss, proliferative retinopathy at baseline, previous photocoagulation | Retinopathy, retinopathy severity, proteinuria, parity, smoking | Retinopathy deterioration |
| Hopp, H[9] | 1995 | Germany | Medical records | Retrospective | 161 | Not specified | Pregnant women with White class C-FR diabetes | None stated | White class F/R | Caesarean |
| Kimmerle, R[10] | 1995 | Germany | Patient notes | Retrospective | 150 | Not specified | All pregnancies with White class F or T, persistent macroproteinuria (>400mg/day or >1+dipstick) or hypertension with creatinine clearance <80mg/mL in the first trimester. Compared to diabetic pregnant women without nephropathy. | Active urine infection or other cause of renal disease in first trimester | Nephropathy | Caesarean, congenital anomaly, SGA, preterm <34/40, perinatal mortality, retinopathy deterioration |
| Axer-Siegel, R[11] | 1996 | Israel | Medical records, biochemistry, fundal photographs | Prospective | 65 | IDDM | All consecutive pregnant women with IDDM. | Women with abortions | Retinopathy | Congenital anomaly, retinopathy deterioration |
| Gordon, M[12] | 1996 | USA | Medical records | Retrospective | 49 | IDDM | Pregnancies complicated by IDDM and diabetic nephropathy. | Miscarriage/termination of pregnancy | Nephropathy | Preterm <34/40 |
| Miodovnik, M[13] | 1996 | USA | Medical records and computerised database | Retrospective | 229 | IDDM | Insulin-dependent diabetics attending clinic with a pregnancy that continued beyond 20 weeks. | Patients with renal transplants | Nephropathy, pre-eclampsia, chronic hypertension, ethnicity, parity | Caesarean, pre-eclampsia, major congenital malformation, LGA, preterm <37/40, preterm <34/40, stillbirth/NND, retinopathy deterioration |
| Lovestam-Adrian, M[14] | 1997 | Sweden | Medical records | Retrospective | 65 | T1DM | All pregnant women with T1DM , compared to non-pregnant women with T1DM | None stated | Retinopathy, pre-eclampsia | Retinopathy deterioration |
| Zhu, L[15] | 1997 | Japan | Medical records | Retrospective | 482 | NIDDM/IDDM | All pregnancies complicated by diabetes mellitus | None stated | Retinopathy, Retinopathy or nephropathy | Caesarean, LGA, preterm <37/40 |
| Hanson, U[16] | 1998 | Sweden | Swedish registry | Prospective | 532 | T1DM | Pregnant women with T1DM | None stated | Retinopathy | Pre-eclampsia |
| Bhattacharyya, A[17] | 1999 | UK | Medical records | Retrospective | 40 | T1/2 DM | Pregnant women with type 1 or 2 diabetes | None stated | Retinopathy, previous photocoagulation | Retinopathy deterioration |
| Biesenbach, G[18] | 1999 | Austria | Patient reports, medical records and biochemistry. | Prospective | 14 | T1DM | Type 1 diabetic pregnant women with macroproteinuria and either low or near-normal creatinine clearance before conception. | 1st trimester abortions | Nephropathy | Progression to nephrotic syndrome |
| Melamed, N[19] | 1999 | Israel | Medical records | Retrospective | 448 | T1/2 DM | Pregnant women with type 1 or 2 diabetes mellitus, compared to pregnant non-diabetic women | Women with multiple fetuses or who delivered before 24 weeks' gestation. | Nephropathy, retinopathy | Preterm <37/40 |
| Biesenbach, G[20] | 2000 | Austria | Medical records | Retrospective | 10 | Not specified | Diabetic women with overt diabetic nephropathy | Macroproteinuria secondary to urinary tract infection or other renal disease | Nephropathy | Caesarean, pre-eclampsia, congenital anomaly, LGA, preterm <34/40 |
| Hiilesmaa, V[21] | 2000 | Finland | Not stated | Retrospective | 683 | T1DM | Pregnan women with pre-existing type I diabetes and pregnancies lasting at least 20 weeks. | None stated | Retinopathy | Pre-eclampsia |
| Ekbom, P[22] | 2001 | Denmark | Patient reports, ophthalmic examinations, biochemistry, medical records | Prospective | 240 | T1DM | Caucasian women with type 1 diabetes attending the obstetric clinic before 17 weeks gestation. | Women with miscarriages at <22 weeks gestation. | Nephropathy | Pre-eclampsia, major congenital anomaly, SGA, preterm <37/40, preterm <34/40, perinatal mortality |
| Lauszus, FF[23] | 2001 | Denmark | Primary data collection | Prospective | 151 | IDDM | Pregnant women with insulin-dependent diabetes, attending the outpatient maternity ward. | Women with insufficient assessment of blood pressure / no first trimester ambulatory blood pressure monitoring. | White class | Pre-eclampsia, pregnancy-induced hypertension |
| Maayah, J[24] | 2001 | Jordan | Medical records | Retrospective | 60 | IDDM | Pregnant insulin-dependent diabetics compared to non-pregnant diabetic controls. | None stated | Retinopathy, retinopathy severity | Retinopathy deterioration |
| McElvy, SS[25] | 2001 | USA | Patient reports, ophthalmic examinations | Prospective | 205 | T1DM | Pregnant women with type 1 diabetes who enrolled before 14 weeks gestation and had at least 2 ophthalmic examinations. | None stated | Retinopathy, retinopathy severity, nephropathy, pre-eclampsia, chronic hypertension, ethnicity, parity | Retinopathy deterioration |
| Temple, RC[26] | 2001 | UK | Patient reports and ophthalmic examinations | Prospective | 179 | T1DM | All type 1 diabetic women with pregnancies progressing beyond 24 gestational weeks | Incomplete data | Smoking | Retinopathy deterioration |
| Lauszus, FF[27] | 2003 | Denmark | Medical records, ophthalmic examinations | Prospective | 142 | T1DM | Pregnant women with type 1 diabetes | Insufficient data | Retinopathy, retinopathy severity | Retinopathy deterioration |
| Campos, MV[28] | 2004 | Portugal | Medical records | Retrospective | 18 | T1DM | Women with type 1 diabetes | None stated | Nephropathy, retinopathy | LGA |
| Larsen, M[29] | 2005 | Finland | Medical records, ophthalmic examinations | Prospective | 45 | T1DM | Pregnant women with type 1 diabetes compared to non-diabetic pregnant women | None stated | Smoking | Retinopathy deterioration |
| Temple, RC[30] | 2006 | UK | Not stated | Prospective | 290 | T1 DM | Pregnant women with type 1 diabetes | None | Microvascular complications | Pre-eclampsia |
| Howarth, C[31] | 2007 | UK | Medical records | Retrospective | 138 | T1DM | Type I diabetic pregnant women with their first viable pregnancies | Pregnant women with multiple fetuses, miscarriages or fetal congenital anomalies | Retinopathy, nephropathy, any diabetic vascular disease | Pre-eclampsia, preterm <37/40 |
| Rahman, W[32] | 2007 | Saudi Arabia | Medical records, ophthalmic examinations | Prospective | 54 | T1DM | Insulin-dependent pregnant diabetics | None stated | Retinopathy, retinopathy severity, previous photocoagulation | Retinopathy deterioration |
| Arun, CS[33] | 2008 | UK | Medical records and ophthalmic records | Retrospective | 59 | T1DM | Type 1 diabetic women who had retinal photographs pre-conception and yearly for 5 years post-conception | None stated | Retinopathy | Retinopathy deterioration |
| Haeri, S[34] | 2008 | USA | Medical records | Prospective | 340 | T1DM | Pregnant women with type 1 diabetes | Pregnancies complicated by pregnancy loss before 20 weeks, major malformations or multiple gestations | Retinopathy and nephropathy | Caesarean, pre-eclampsia, LGA, SGA, preterm <37/40, preterm <34/40, perinatal mortality |
| Dodesini, AR[35] | 2009 | Italy | Medical records and laboratory data | Retrospective | 29 | T1DM | Pregnant women with type 1 diabetes | Women with previous caesarean section | Retinopathy and nephropathy | Caesarean |
| Nielsen, LR[36] | 2009 | Denmark | Medical records, biochemistry records | Prospective | 117 | T1DM | Singleton pregnancies in Danish-speaking Caucasian women with type 1 diabetes, referred before 14 weeks gestational age | Women with pregnany loss before 22 completed gestational weeks, subsequent pregnancies in the same women during the study period, women with Addison's disease and inflammatory bowel disease | Nephropathy | Pre-eclampsia, LGA, SGA, preterm <37/40, preterm <34/40, perinatal mortality |
| Lepercq, J[37] | 2010 | France | Medical records | Prospective | 209 | T1DM | Consecutive nulliparous women with type 1 diabetes and a singleton pregnancy who delivered a liveborn infant after 22 weeks gestation. | Major congenital malformations of the fetus | Nephropathy | Caesarean |
| Rasmussen, KL[38] | 2010 | Denmark | Medical records | Retrospective | 110 | T2DM | Singleton pregnancies in women with type 2 diabetes, referred before 20 weeks gestation and delivered after 22 weeks. | Subsequent pregnancies during the study period in the same women, women whose medical record could not be identified, women with less than 2 ophthalmic examinations | Retinopathy, macular oedema, albuminuria, pre-eclampsia, chronic hypertension, smoking | Retinopathy deterioration |
| Ringholm, L[39] | 2011 | Denmark | Not stated | Not stated | 84 | T1DM | Pregnant women with type 1 diabetes | None stated | Nephropathy, retinopathy | Pre-eclampsia |
| Ringholm, L[40] | 2011 | Denmark | Medical records, ophthalmic examinations | Prospective | 88 | T1DM | Pregnant women with type 1 diabetes, referred prior to 14 weeks gestation | None stated | Retinopathy, retinopathy severity, nephropathy, chronic hypertension | Retinopathy deterioration |
| Young, EC[41] | 2011 | Brazil | Medical records | Prospective | 60 | T1/2 DM | Pregnant women with diabetes mellitus, starting prenatal care before 12 weeks gestation | None stated | Nephropathy | Pre-eclampsia, preterm <37/40 |
| Abe, Y[42] | 2012 | Japan | Not stated | Retrospective | 462 | T1/2 DM | Women with pre-gestational diabetes and singleton pregnancies | None stated | Pre-gestational glycated hemoglobin, BMI, retinopathy, nephropathy, type of diabetes | Pregnancy-induced hypertension |
| Bell, R[43] | 2012 | Portugal | Registers of congenital anomaly and diabetes in pregnancy | Retrospective | 1677 | T1/2 DM | Singleton pregnancies in women with diabetes and live birth, fetal death ≥20 weeks gestation or termination for fetal anomaly. | None stated | Retinopathy, nephropathy, neuropathy | Congenital anomaly |
| Themeli, Y[44] | 2012 | Albania | Medical records, biochemistry | Prospective | 80 | T1DM | Pregnant women with type 1 diabetes attending before 17 weeks gestation with a living fetus | Women with miscarriages at <22 weeks gestation. | Nephropathy | Pre-eclampsia, major congenital malformation, SGA, preterm <37/40, preterm <34/40, perinatal mortality |
| Damm, JA[45] | 2013 | Denmark | Not stated | Retrospective | 41 | T1/2 DM | Singleton pregnancies in women with pre-existing diabetes and with a living fetus beyond 22 completed gestational weeks. | None stated | Nephropathy | LGA, SGA, preterm <37/40, preterm <34/40, NICU admission |
| Patel, N[46] | 2013 | UK | Not stated | Retrospective | 37 | T1DM | Pregnant women with type 1 diabetes mellitus on continuous subcutaneous insulin infusions | None stated | Diabetic microvascular complication | Pre-eclampsia |
| Castiglioni, MT[47] | 2014 | Italy | Medical records | Prospective | 291 | T1DM | Pregnant women with type 1 diabetes | Type 2 diabetes, overt nephropathy, multiple pregnancy. | Retinopathy | Pre-eclampsia |
| Herman, M[48] | 2014 | Croatia | Medical records | Retrospective | 396 | T1DM | Pregnant women with type 1 diabetes | None stated | Nephropathy | Perinatal mortality |
| Egan, AM[49] | 2015 | Ireland | Medical database | Retrospective | 185 | T1/T2DM | Pregnant women with pre-gestational diabetes having at least 2 retinal screens in different trimesters. | Women with less than 2 retinal examinations in pregnancy. Women with pregnancy losses before 22 completed gestational weeks. | Retinopathy, diabetes type, pre-eclampsia, ethnicity | Retinopathy deterioration |
| Klemetti, MM[50] | 2016 | Finland | Obstetric records | Retrospective | 1094 | T1DM | Consecutive pregnant women with type 1 diabetes and a singleton childbirth | If one woman had more than one childbirth during the time period, all but the latest one was excluded. | Nephropathy, retinopathy | Caesarean, pre-eclampsia, preterm <37/40, perinatal mortality, NICU admission |
| Toda, J[51] | 2016 | Japan | Medical records | Retrospective | 93 | DM | Pregnant women with type 1 or 2 diabetes | Patients whose ophthamic records could not be found. | Retinopathy, previous photocoagulation, diabetes type | Retinopathy deterioration |
| Durackova, L[52] | 2017 | Slovakia | Hospital database | Retrospective | 118 | T1 DM | Pregnant women with type 1 diabetes | None stated | Diabetic vasculopathy | Pre-eclampsia, preterm birth, mode of birth, birthweight (SGA/LGA), apgar score, neonatal morbidity, perinatal mortality. |
| Gutaj, P[53] | 2017 | Poland | Primary data collection | Prospective | 165 | T1 DM | Pregnant women with type 1 diabetes | None stated | Diabetic vasculopathy | Gestational hypertension, pre-eclampsia. |
| Norgaard, SK[54] | 2018 | Denmark | Not stated | Prospective | 494 | T1/2 DM | Pregnant women with type 1 or 2 diabetes | Previous bariatric surgery, severe concomitant illness, subsequent pregnancies during the study period. | Retinopathy, nephropathy | Pregnancy-related hypertensive disorders |
| Samii, L[55] | 2019 | Canada | Medical records | Retrospective | 232 | T1 DM | Pregnant women with type 1 diabetes | Moved out of area, unknown gestation, delivery after the study period | Vascular complications of diabetes | Congenital anomaly |
| Bourry, J[56] | 2021 | France | Medical records | Retrospective | 499 | T1 DM | Pregnant women with type 1 diabetes | Women with spontaneous/induced abortion. | Retinopathy, nephropathy, nulliparity, photocoagulation. | New onset or progression of retinopathy. |
| Abbreviations: <34/40: Birth prior to 34 completed gestational weeks, <37/40: Birth prior to 37 completed gestational weeks, GDM: Gestational diabetes mellitus, IDDM: Insulin-dependent diabetes mellitus, LGA: Large for gestational age neonate, NICU: Neonatal intensive care unit, NIDDM: Non insulin-dependent diabetes mellitus, SGA: Small for gestational age neonate, T1DM: Type 1 diabetes mellitus, T2DM: Type 2 diabetes mellitus, White class D (Diabetes developed at <10 years age, or duration of diabetes >20 years, or background retinopathy, or chronic hypertension), White class F (Diabetes with nephropathy), White class R (Diabetes with proliferative retinopathy or vitreous haemorrhage). | | | | | | | | | | |

References

1. Garner PR, D'Alton ME, Dudley DK, Huard P, Hardie M: **Preeclampsia in diabetic pregnancies**. *Am J Obstet Gynecol* 1990, **163**(2):505-508.

2. Schoetzau A, Hillebrand B: **Neonatal morbidity in infants of diabetic mothers** *Z Geburtshilfe Perinatol* 1990, **194**(2):58-64.

3. Hoshi J, Nishida H, Takahashi N, Kabe K, Watanabe Y, Arai T, Yamada T, Yamaguchi K, Sakamoto S: **Perinatal morbidity of infants of diabetic mothers**. *Acta Paediatr Jpn* 1991, **33**(2):159-165.

4. Ayed S, Jeddi A, Daghfous F, El Euch M, Ben Osman N, Marrakchi S, Nacef L: **Progressive aspects of diabetic retinopathy during pregnancy**. *J Fr Ophtalmol* 1992, **15**(8-9):474-477.

5. Rosenn B, Miodovnik M, Kranias G, Khoury J, Combs CA, Mimouni F, Siddiqi TA, Lipman MJ: **Progression of diabetic retinopathy in pregnancy: association with hypertension in pregnancy**. *Am J Obstet Gynecol* 1992, **166**(4):1214-1218.

6. Combs CA, Rosenn B, Kitzmiller JL, Khoury JC, Wheeler BC, Miodovnik M: **Early-pregnancy proteinuria in diabetes related to preeclampsia**. *Obstet Gynecol* 1993, **82**(5):802-807.

7. Chen HC, Newsom RS, Patel V, Cassar J, Mather H, Kohner EM: **Retinal blood flow changes during pregnancy in women with diabetes**. *Invest Ophthalmol Vis Sci* 1994, **35**(8):3199-3208.

8. Chew EY, Mills JL, Metzger BE, Remaley NA, Jovanovic-Peterson L, Knopp RH, Conley M, Rand L, Simpson JL, Holmes LB *et al*: **Metabolic control and progression of retinopathy. The Diabetes in Early Pregnancy Study. National Institute of Child Health and Human Development Diabetes in Early Pregnancy Study**. *Diabetes Care* 1995, **18**(5):631-637.

9. Hopp H, Vollert W, Ebert A, Weitzel H, Glockner E, Jahrig D: **Diabetic retinopathy and nephropathy - Complications during pregnancy and delivery**. *Geburtshilfe und Frauenheilkunde* 1995, **55**(5):275-279.

10. Kimmerle R, Zass R, Cupisti S, Somville T, Bender R, Pawlowski B, Berger M: **Pregnancies in women with diabetic nephropathy: Long-term outcome for mother and child**. *Diabetologia* 1995, **38**(2):227-235.

11. Axer-Siegel R, Hod M, Fink-Cohen S, Kramer M, Weinberger D, Schindel B, Yassur Y: **Diabetic retinopathy during pregnancy**. *Ophthalmology* 1996, **103**(11):1815-1819.

12. Gordon M, Landon M, Samuels P, Hissrich S, Gabbe S: **Perinatal outcome and long-term follow-up associated with modern management of diabetic nephropathy**. *Obstetrics and Gynecology* 1996, **87**(3):401-409.

13. Miodovnik M, Rosenn B, Khoury J, Grigsby J, Siddiqi T, Langer O, Roberts J, McDonough P, Gabbe S, Spinnato J: **Does pregnancy increase the risk for development and progression of diabetic nephropathy?** *American Journal of Obstetrics and Gynecology* 1996, **174**(4):1180-1191.

14. Lovestam-Adrian M, Agardh C, Aberg A, Agardh E: **Pre-eclampsia is a potent risk factor for deterioration of retinopathy during pregnancy in Type 1 diabetic patients**. *Diabetic Medicine* 1997, **14**(12):1059-1065.

15. Zhu L, Nakabayashi M, Takeda Y: **Statistical analysis of perinatal outcomes in pregnancy complicated with diabetes mellitus**. *J Obstet Gynaecol Res* 1997, **23**(6):555-563.

16. Hanson U, Persson B: **Epidemiology of pregnancy-induced hypertension and preeclampsia in type 1 (insulin-dependent) diabetic pregnancies in Sweden**. *Acta Obstet Gynecol Scand* 1998, **77**(6):620-624.

17. Bhattacharyya A, Vice PA: **Insulin lispro, pregnancy, and retinopathy**. *Diabetes Care* 1999, **22**(12):2101-2104.

18. Biesenbach G, Grafinger P, Stoger H, Zazgornik J: **How pregnancy influences renal function in nephropathic type 1 diabetic women depends on their pre-conceptional creatinine clearance**. *Journal of Nephrology Nursing* 1999, **12**(1):41-46.

19. Melamed N, Chen R, Soiberman U, Ben-Haroush A, Hod M, Yogev Y: **Spontaneous and indicated preterm delivery in pregestational diabetes mellitus: etiology and risk factors**. *Arch Gynecol Obstet* 2008, **278**(2):129-134.

20. Biesenbach G, Grafinger P, Zazgornik J, Helmut, Stoger: **Perinatal complications and three-year follow up of infants of diabetic mothers with diabetic nephropathy stage IV**. *Ren Fail* 2000, **22**(5):573-580.

21. Hiilesmaa V, Suhonen L, Teramo K: **Glycaemic control is associated with pre-eclampsia but not with pregnancy-induced hypertension in women with type I diabetes mellitus.** *Diabetologia* 2000, **43**(12):1534-1539.

22. Ekbom P, Damm P, Feldt-Rasmussen B, Feldt-Rasmussen U, Molvig J, Mathiesen E: **Pregnancy outcome in type 1 diabetic women with microalbuminuria** *Diabetes care* 2001, **24**(10):1739-1744.

23. Lauszus FF, Rasmussen OW, Lousen T, Klebe TM, Klebe JG: **Ambulatory blood pressure as predictor of preeclampsia in diabetic pregnancies with respect to urinary albumin excretion rate and glycemic regulation**. *Acta Obstet Gynecol Scand* 2001, **80**(12):1096-1103.

24. Maayah J, Shammas A, Haddadin A: **Effect of pregnancy on diabetic retinopathy**. *Bahrain Medical Bulletin* 2001, **23**(4):163-165.

25. McElvy SS, Demarini S, Miodovnik M, Khoury JC, Rosenn B, Tsang RC: **Fetal weight and progression of diabetic retinopathy**. *Obstet Gynecol* 2001, **97**(4):587-592.

26. Temple RC, Aldridge VA, Sampson MJ, Greenwood RH, Heyburn PJ, Glenn A: **Impact of pregnancy on the progression of diabetic retinopathy in Type 1 diabetes**. *Diabet Med* 2001, **18**(7):573-577.

27. Lauszus F, Klebe J, Bek T, Flyvbjerg A: **Increased serum IGF-I during pregnancy is associated with progression of diabetic retinopathy**. *Diabetes* 2003, **52**(3):852-856.

28. Campos MV, Ruas L, Paiva S, Leitao P, Lobo C, Marta E, Sobral E, Cravo A, Carvalheiro M: **Fetal growth and glycemic control in type 1 diabetes pregnancy**. *Acta Med Port* 2004, **17**(2):167-172.

29. Larsen M, Colmorn LB, Bonnelycke M, Kaaja R, Immonen I, Sander B, Loukovaara S: **Retinal artery and vein diameters during pregnancy in diabetic women**. *Invest Ophthalmol Vis Sci* 2005, **46**(2):709-713.

30. Temple RC, Aldridge V, Stanley K, Murphy HR: **Glycaemic control throughout pregnancy and risk of pre-eclampsia in women with type I diabetes**. *BJOG* 2006, **113**(11):1329-1332.

31. Howarth C, Gazis A, James D: **Associations of Type 1 diabetes mellitus, maternal vascular disease and complications of pregnancy**. *Diabet Med* 2007, **24**(11):1229-1234.

32. Rahman W, Rahman FZ, Yassin S, Al-Suleiman SA, Rahman J: **Progression of retinopathy during pregnancy in type 1 diabetes mellitus**. *Clin Exp Ophthalmol* 2007, **35**(3):231-236.

33. Arun CS, Taylor R: **Influence of pregnancy on long-term progression of retinopathy in patients with type 1 diabetes**. *Diabetologia* 2008, **51**(6):1041-1045.

34. Haeri S, Khoury J, Kovilam O, Miodovnik M: **The association of intrauterine growth abnormalities in women with type 1 diabetes mellitus complicated by vasculopathy**. *Am J Obstet Gynecol* 2008, **199**(3):278 e271-275.

35. Dodesini A, Maffeis A, Corsi A, Benvenuto A, Ciriello E, Lepore G, Strobelt N, Nosari I, Frigerio L, Trevisan R: **In type 1 diabetic women retinopathy and nephropathy are predictors of caesarean delivery independently of glucose control** *Diabetes* 2009, **58**.

36. Nielsen LR, Damm P, Mathiesen ER: **Improved pregnancy outcome in type 1 diabetic women with microalbuminuria or diabetic nephropathy: effect of intensified antihypertensive therapy?** *Diabetes Care* 2009, **32**(1):38-44.

37. Lepercq J, Le Meaux JP, Agman A, Timsit J: **Factors associated with cesarean delivery in nulliparous women with type 1 diabetes**. *Obstet Gynecol* 2010, **115**(5):1014-1020.

38. Rasmussen KL, Laugesen CS, Ringholm L, Vestgaard M, Damm P, Mathiesen ER: **Progression of diabetic retinopathy during pregnancy in women with type 2 diabetes**. *Diabetologia* 2010, **53**(6):1076-1083.

39. Ringholm L, Pedersen-Bjergaard U, Thorsteinsson B, Boomsma F, Damm P, Mathiesen E: **Higher levels of Atrial Natriuretic Peptide (ANP) are present in early pregnancy in type 1 diabetic women developing preeclampsia** *, September 2011, vol/is 54/* 2011:S485.

40. Ringholm L, Vestgaard M, Laugesen CS, Juul A, Damm P, Mathiesen ER: **Pregnancy-induced increase in circulating IGF-I is associated with progression of diabetic retinopathy in women with type 1 diabetes**. *Growth Horm IGF Res* 2011, **21**(1):25-30.

41. Young EC, Pires ML, Marques LP, de Oliveira JE, Zajdenverg L: **Effects of pregnancy on the onset and progression of diabetic nephropathy and of diabetic nephropathy on pregnancy outcomes**. *Diabetes Metab Syndr* 2011, **5**(3):137-142.

42. Abe Y, Mitani M, Fukazawa Y, Konno J, Makino Y, Matsuda Y: **Pregnancy induced hypertension in women with pregestational diabetes mellitus**. *J Paediatr Child Health* 2012, **48**(117):1034-4810.

43. Bell R, Glinianaia SV, Tennant PW, Bilous RW, Rankin J: **Peri-conception hyperglycaemia and nephropathy are associated with risk of congenital anomaly in women with pre-existing diabetes: a population-based cohort study**. *Diabetologia* 2012.

44. Themeli Y, Bajrami V, Zaimi K, Mustafaraj K, Lulo J, Peci E, Gjoshe J, Shtylla A, Barbullushi M, Idrizi A *et al*: **Diabetic nephropathy in pregnant women with type 1 diabetes mellitus**. *Giornale Italiano di Ostetricia e Ginecologia* 2012, **34**(1):323-327.

45. Damm JA, Asbjornsdottir B, Callesen NF, Mathiesen JM, Ringholm L, Pedersen BW, Mathiesen ER: **Diabetic nephropathy and microalbuminuria in pregnant women with type 1 and type 2 diabetes: prevalence, antihypertensive strategy, and pregnancy outcome**. *Diabetes Care* 2013, **36**(11):3489-3494.

46. Patel N, Brackenridge A, Kanji A, Pasupathy D, Rajasingam D: **Micro-vascular disease at booking in T1DM and associated risk of developing pre-eclampsia**. *Archives of Disease in Childhood: Fetal and Neonatal Edition* 2013, **98**:1359-2998.

47. Castiglioni MT, Valsecchi L, Cavoretto P, Pirola S, Di Piazza L, Maggio L, Caretto A, Garito TS, Rosa S, Scavini M: **The risk of preeclampsia beyond the first pregnancy among women with type 1 diabetes parity and preeclampsia in type 1 diabetes**. *Pregnancy Hypertens* 2014, **4**(1):34-40.

48. Herman M, Djelmis J, Ivanisevic M, Blajic J, Starcevic V: **Pregnancy outcome of mothers with diabetic nephropathy**. *Journal of Maternal-Fetal and Neonatal Medicine* 2014, **27**:266-267.

49. Egan AM, McVicker L, Heerey A, Carmody L, Harney F, Dunne FP: **Diabetic retinopathy in pregnancy: a population-based study of women with pregestational diabetes**. *J Diabetes Res* 2015, **2015**:310239.

50. Klemetti MM, Laivuori H, Tikkanen M, Nuutila M, Hiilesmaa V, Teramo K: **White's classification and pregnancy outcome in women with type 1 diabetes: a population-based cohort study**. *Diabetologia* 2016, **59**(1):92-100.

51. Toda J, Kato S, Sanaka M, Kitano S: **The effect of pregnancy on the progression of diabetic retinopathy**. *Jpn J Ophthalmol* 2016, **60**(6):454-458.

52. Durackova L, Kristufkova A, Korbel M: **Pregnancy and neonatal outcomes in women with type 1 diabetes mellitus**. *Bratisl Med J* 2017, **118**(1):56-60.

53. Gutaj P, Zawiejska A, Mantaj U, Ożegowska E: **Determinants of preeclampsia in women with type 1 diabetes**. *Acta Diabetol* 2017, **54**:1115-1121.

54. Norgaard SK, Vestgaard MJ, Jorgensen IL, Asbjornsdottir B, Ringholm L, McIntyre HD, Damm P, Mathiesen ER: **Diastolic blood pressure is a potentially modifiable risk factor for preeclampsia in women with pre-existing diabetes**. *Diabetes Res Clin Pract* 2018, **138**:229-237.

55. Samii L, Kallas-Koeman M, Donovan LE, Lodha A, Crawford S, Butalia S: **The association between vascular complications during pregnancy in women with Type 1 diabetes and congenital malformations**. *Diabet Med* 2019, **36**(2):237-242.

56. Bourry J, Courteville H, Ramdane N, Drumez E, Duhamel A, Subtil D, Deruelle P, Vambergue A: **Progression of Diabetic Retinopathy and Predictors of Its Development and Progression During Pregnancy in Patients With Type 1 Diabetes: A Report of 499 Pregnancies**. *Diabetes Care* 2021, **44**(1):181-187.
