# Supplementary material for: Adverse pregnancy outcomes in women with diabetes-related microvascular disease and risks of disease progression in pregnancy: A systematic review and meta-analysis
Source: PLoS Med. 2021 Nov 22;18(11):e1003856. doi: 10.1371/journal.pmed.1003856 (PMC8654151; doi:10.1371/journal.pmed.1003856)
Supplement: S5 Appendix — (DOCX) [file pmed.1003856.s005.docx]

**S5 Appendix: Scores given to included studies using the Newcastle-Ottawa scale for assessment of potential bias.**

| Study | | Newcastle-Ottawa Scale score | | | |
| --- | --- | --- | --- | --- | --- |
| **Author** | **Year** | **Selection (/****)** | **Comparability (/**)** | **Outcome/ Exposure (/***)** | **Overall** |
| Garner, PR[1] | 1990 | Low (****) | High (-) | Low (***) | High |
| Schoetzau, A[2] | 1990 | Low (****) | High (-) | Low (***) | High |
| Hoshi, J[3] | 1991 | Low (****) | High (-) | Low (***) | High |
| Ayed, S[4] | 1992 | Medium (**) | High (-) | Medium (**) | High |
| Rosenn, B[5] | 1992 | Low (****) | High (-) | Medium (**) | High |
| Combs, CA[6] | 1993 | Low (****) | Low (**) | Medium (**) | Medium |
| Chen, HC[7] | 1994 | Medium (**) | High (-) | Medium (**) | High |
| Chew, EY[8] | 1995 | Medium (**) | Low (**) | Medium (**) | Medium |
| Hopp, H[9] | 1995 | Low (****) | High (-) | Low (***) | High |
| Kimmerle, R[10] | 1995 | Low (****) | Low (**) | Low (***) | Low |
| Axer-Siegel, R[11] | 1996 | Low (****) | Medium (*) | Low (***) | Medium |
| Gordon, M[12] | 1996 | Low (****) | High (-) | Medium (**) | High |
| Miodovnik, M[13] | 1996 | Low (****) | Low (**) | Medium (**) | Medium |
| Lovestam-Adrian, M[14] | 1997 | Low (****) | Low (**) | Low (***) | Low |
| Zhu, L[15] | 1997 | Low (****) | Low (**) | Low (***) | Low |
| Hanson, U[16] | 1998 | Low (****) | High (-) | Low (***) | High |
| Bhattacharyya, A[17] | 1999 | Medium (**) | High (-) | Medium (**) | High |
| Biesenbach, G[18] | 1999 | Medium (**) | High (-) | Low (***) | High |
| Melamed, N[19] | 1999 | Low (****) | High (-) | Low (***) | High |
| Biesenbach, G[20] | 2000 | Low (****) | High (-) | Low (***) | High |
| Hiilesmaa, V[21] | 2000 | Medium (***) | Low (**) | Low (***) | Medium |
| Ekbom, P[22] | 2001 | Medium (***) | Medium (*) | Low (***) | Medium |
| Lauszus, FF[23] | 2001 | Low (****) | Low (**) | Low (***) | Low |
| Maayah, J[24] | 2001 | Low (****) | High (-) | Low (***) | High |
| McElvy, SS[25] | 2001 | Low (****) | Medium (*) | Low (***) | Medium |
| Temple, RC[26] | 2001 | Medium (***) | High (-) | Low (***) | High |
| Lauszus, FF[27] | 2003 | Medium (***) | High (-) | Low (***) | High |
| Campos, MV[28] | 2004 | Medium (**) | High (-) | Low (***) | High |
| Larsen, M[29] | 2005 | Low (****) | Medium (*) | Low (***) | Medium |
| Temple, RC[30] | 2006 | Low (****) | Low (**) | Low (***) | Low |
| Howarth, C[31] | 2007 | Low (****) | Low (**) | Low (***) | Low |
| Rahman, W[32] | 2007 | Medium (***) | High (-) | Low (***) | High |
| Arun, CS[33] | 2008 | Medium (***) | High (-) | Low (***) | High |
| Haeri, S[34] | 2008 | Low (****) | Medium (*) | Low (***) | Medium |
| Dodesini, AR[35] | 2009 | Low (****) | Low (**) | Low (***) | Low |
| Nielsen, LR[36] | 2009 | Medium (***) | Medium (*) | Low (***) | Medium |
| Lepercq, J[37] | 2010 | Medium (***) | Low (**) | Low (***) | Medium |
| Rasmussen, KL[38] | 2010 | Medium (***) | High (-) | Medium (**) | High |
| Ringholm, L[39] | 2011 | Low (****) | High (-) | Medium (**) | High |
| Ringholm, L[40] | 2011 | Medium (***) | Low (**) | Low (***) | Medium |
| Young, EC[41] | 2011 | Medium (**) | High (-) | Medium (**) | High |
| Abe, Y[42] | 2012 | Low (****) | High (-) | Low (***) | High |
| Bell, R[43] | 2012 | Low (****) | Low (**) | Low (***) | Low |
| Themeli, Y[44] | 2012 | Medium (***) | Low (**) | Low (***) | Medium |
| Damm, JA[45] | 2013 | Low (****) | High (-) | Low (***) | High |
| Patel, N[46] | 2013 | Low (****) | Low (**) | Medium (**) | Medium |
| Castiglioni, MT[47] | 2014 | Low (****) | Low (**) | Low (***) | Low |
| Herman, M[48] | 2014 | Medium (***) | High (-) | Medium (**) | High |
| Egan, AM[49] | 2015 | Medium (***) | High (-) | Medium (**) | High |
| Klemetti, MM[50] | 2016 | Medium (***) | Low (**) | Low (***) | Medium |
| Toda, J[51] | 2016 | Medium (***) | High (-) | Low (***) | High |
| Durackova, L[52] | 2017 | Low (****) | High (-) | Low (***) | High |
| Gutaj, P[53] | 2017 | Low (****) | Low (**) | Low (***) | Low |
| Norgaard, SK[54] | 2018 | Low (****) | Low (**) | Low (***) | Low |
| Samii, L[55] | 2019 | Low (****) | Low (**) | Low (***) | Low |
| Bourry, J[56] | 2021 | Low (****) | Low (**) | Low (***) | Low |

References

1. Garner PR, D'Alton ME, Dudley DK, Huard P, Hardie M. Preeclampsia in diabetic pregnancies. Am J Obstet Gynecol. 1990;163(2):505-8. Epub 1990/08/01. PubMed PMID: 2386136.

2. Schoetzau A, Hillebrand B. Neonatal morbidity in infants of diabetic mothers Z Geburtshilfe Perinatol. 1990;194(2):58-64.

3. Hoshi J, Nishida H, Takahashi N, Kabe K, Watanabe Y, Arai T, et al. Perinatal morbidity of infants of diabetic mothers. Acta Paediatr Jpn. 1991;33(2):159-65. Epub 1991/04/01. PubMed PMID: 1957639.

4. Ayed S, Jeddi A, Daghfous F, El Euch M, Ben Osman N, Marrakchi S, et al. Progressive aspects of diabetic retinopathy during pregnancy. J Fr Ophtalmol. 1992;15(8-9):474-7.

5. Rosenn B, Miodovnik M, Kranias G, Khoury J, Combs CA, Mimouni F, et al. Progression of diabetic retinopathy in pregnancy: association with hypertension in pregnancy. Am J Obstet Gynecol. 1992;166(4):1214-8. Epub 1992/04/01. PubMed PMID: 1566772.

6. Combs CA, Rosenn B, Kitzmiller JL, Khoury JC, Wheeler BC, Miodovnik M. Early-pregnancy proteinuria in diabetes related to preeclampsia. Obstet Gynecol. 1993;82(5):802-7. Epub 1993/11/01. PubMed PMID: 8414328.

7. Chen HC, Newsom RS, Patel V, Cassar J, Mather H, Kohner EM. Retinal blood flow changes during pregnancy in women with diabetes. Invest Ophthalmol Vis Sci. 1994;35(8):3199-208. Epub 1994/07/01. PubMed PMID: 8045714.

8. Chew EY, Mills JL, Metzger BE, Remaley NA, Jovanovic-Peterson L, Knopp RH, et al. Metabolic control and progression of retinopathy. The Diabetes in Early Pregnancy Study. National Institute of Child Health and Human Development Diabetes in Early Pregnancy Study. Diabetes Care. 1995;18(5):631-7. Epub 1995/05/01. PubMed PMID: 8586000.

9. Hopp H, Vollert W, Ebert A, Weitzel H, Glockner E, Jahrig D. Diabetic retinopathy and nephropathy - Complications during pregnancy and delivery. Geburtshilfe und Frauenheilkunde. 1995;55(5):275-9.

10. Kimmerle R, Zass R, Cupisti S, Somville T, Bender R, Pawlowski B, et al. Pregnancies in women with diabetic nephropathy: Long-term outcome for mother and child. Diabetologia. 1995;38(2):227-35.

11. Axer-Siegel R, Hod M, Fink-Cohen S, Kramer M, Weinberger D, Schindel B, et al. Diabetic retinopathy during pregnancy. Ophthalmology. 1996;103(11):1815-9. Epub 1996/11/01. doi: 10.1016/s0161-6420(96)30421-1. PubMed PMID: 8942876.

12. Gordon M, Landon M, Samuels P, Hissrich S, Gabbe S. Perinatal outcome and long-term follow-up associated with modern management of diabetic nephropathy. Obstetrics and Gynecology. 1996;87(3):401-9.

13. Miodovnik M, Rosenn B, Khoury J, Grigsby J, Siddiqi T, Langer O, et al. Does pregnancy increase the risk for development and progression of diabetic nephropathy? American Journal of Obstetrics and Gynecology. 1996;174(4):1180-91.

14. Lovestam-Adrian M, Agardh C, Aberg A, Agardh E. Pre-eclampsia is a potent risk factor for deterioration of retinopathy during pregnancy in Type 1 diabetic patients. Diabetic Medicine. 1997;14(12):1059-65.

15. Zhu L, Nakabayashi M, Takeda Y. Statistical analysis of perinatal outcomes in pregnancy complicated with diabetes mellitus. J Obstet Gynaecol Res. 1997;23(6):555-63. Epub 1998/01/20. PubMed PMID: 9433048.

16. Hanson U, Persson B. Epidemiology of pregnancy-induced hypertension and preeclampsia in type 1 (insulin-dependent) diabetic pregnancies in Sweden. Acta Obstet Gynecol Scand. 1998;77(6):620-4. Epub 1998/08/04. PubMed PMID: 9688239.

17. Bhattacharyya A, Vice PA. Insulin lispro, pregnancy, and retinopathy. Diabetes Care. 1999;22(12):2101-4. Epub 1999/12/10. PubMed PMID: 10587864.

18. Biesenbach G, Grafinger P, Stoger H, Zazgornik J. How pregnancy influences renal function in nephropathic type 1 diabetic women depends on their pre-conceptional creatinine clearance. Journal of Nephrology Nursing. 1999;12(1):41-6.

19. Melamed N, Chen R, Soiberman U, Ben-Haroush A, Hod M, Yogev Y. Spontaneous and indicated preterm delivery in pregestational diabetes mellitus: etiology and risk factors. Arch Gynecol Obstet. 2008;278(2):129-34. Epub 2008/01/15. doi: 10.1007/s00404-007-0541-z. PubMed PMID: 18193440.

20. Biesenbach G, Grafinger P, Zazgornik J, Helmut, Stoger. Perinatal complications and three-year follow up of infants of diabetic mothers with diabetic nephropathy stage IV. Ren Fail. 2000;22(5):573-80. Epub 2000/10/21. PubMed PMID: 11041289.

21. Hiilesmaa V, Suhonen L, Teramo K. Glycaemic control is associated with pre-eclampsia but not with pregnancy-induced hypertension in women with type I diabetes mellitus. Diabetologia. 2000;43(12):1534-9.

22. Ekbom P, Damm P, Feldt-Rasmussen B, Feldt-Rasmussen U, Molvig J, Mathiesen E. Pregnancy outcome in type 1 diabetic women with microalbuminuria Diabetes care. 2001;24(10):1739-44.

23. Lauszus FF, Rasmussen OW, Lousen T, Klebe TM, Klebe JG. Ambulatory blood pressure as predictor of preeclampsia in diabetic pregnancies with respect to urinary albumin excretion rate and glycemic regulation. Acta Obstet Gynecol Scand. 2001;80(12):1096-103. Epub 2002/02/16. PubMed PMID: 11846705.

24. Maayah J, Shammas A, Haddadin A. Effect of pregnancy on diabetic retinopathy. Bahrain Medical Bulletin. 2001;23(4):163-5.

25. McElvy SS, Demarini S, Miodovnik M, Khoury JC, Rosenn B, Tsang RC. Fetal weight and progression of diabetic retinopathy. Obstet Gynecol. 2001;97(4):587-92. Epub 2001/03/29. PubMed PMID: 11275032.

26. Temple RC, Aldridge VA, Sampson MJ, Greenwood RH, Heyburn PJ, Glenn A. Impact of pregnancy on the progression of diabetic retinopathy in Type 1 diabetes. Diabet Med. 2001;18(7):573-7. Epub 2001/09/13. PubMed PMID: 11553188.

27. Lauszus F, Klebe J, Bek T, Flyvbjerg A. Increased serum IGF-I during pregnancy is associated with progression of diabetic retinopathy. Diabetes. 2003;52(3):852-6.

28. Campos MV, Ruas L, Paiva S, Leitao P, Lobo C, Marta E, et al. Fetal growth and glycemic control in type 1 diabetes pregnancy. Acta Med Port. 2004;17(2):167-72. Epub 2005/06/01. PubMed PMID: 15921648.

29. Larsen M, Colmorn LB, Bonnelycke M, Kaaja R, Immonen I, Sander B, et al. Retinal artery and vein diameters during pregnancy in diabetic women. Invest Ophthalmol Vis Sci. 2005;46(2):709-13. Epub 2005/01/27. doi: 10.1167/iovs.04-0604. PubMed PMID: 15671303.

30. Temple RC, Aldridge V, Stanley K, Murphy HR. Glycaemic control throughout pregnancy and risk of pre-eclampsia in women with type I diabetes. BJOG. 2006;113(11):1329-32. Epub 2006/09/29. doi: 10.1111/j.1471-0528.2006.01071.x. PubMed PMID: 17004981.

31. Howarth C, Gazis A, James D. Associations of Type 1 diabetes mellitus, maternal vascular disease and complications of pregnancy. Diabet Med. 2007;24(11):1229-34. Epub 2007/08/30. doi: 10.1111/j.1464-5491.2007.02254.x. PubMed PMID: 17725628.

32. Rahman W, Rahman FZ, Yassin S, Al-Suleiman SA, Rahman J. Progression of retinopathy during pregnancy in type 1 diabetes mellitus. Clin Exp Ophthalmol. 2007;35(3):231-6. Epub 2007/04/14. doi: 10.1111/j.1442-9071.2006.01413.x. PubMed PMID: 17430509.

33. Arun CS, Taylor R. Influence of pregnancy on long-term progression of retinopathy in patients with type 1 diabetes. Diabetologia. 2008;51(6):1041-5. Epub 2008/04/09. doi: 10.1007/s00125-008-0994-z. PubMed PMID: 18392803.

34. Haeri S, Khoury J, Kovilam O, Miodovnik M. The association of intrauterine growth abnormalities in women with type 1 diabetes mellitus complicated by vasculopathy. Am J Obstet Gynecol. 2008;199(3):278 e1-5. Epub 2008/09/06. doi: 10.1016/j.ajog.2008.06.066. PubMed PMID: 18771982.

35. Dodesini A, Maffeis A, Corsi A, Benvenuto A, Ciriello E, Lepore G, et al. In type 1 diabetic women retinopathy and nephropathy are predictors of caesarean delivery independently of glucose control Diabetes. 2009;58.

36. Nielsen LR, Damm P, Mathiesen ER. Improved pregnancy outcome in type 1 diabetic women with microalbuminuria or diabetic nephropathy: effect of intensified antihypertensive therapy? Diabetes Care. 2009;32(1):38-44. Epub 2008/10/24. doi: 10.2337/dc08-1526. PubMed PMID: 18945922; PubMed Central PMCID: PMCPMC2606826.

37. Lepercq J, Le Meaux JP, Agman A, Timsit J. Factors associated with cesarean delivery in nulliparous women with type 1 diabetes. Obstet Gynecol. 2010;115(5):1014-20. Epub 2010/04/23. doi: 10.1097/AOG.0b013e3181d992ab. PubMed PMID: 20410777.

38. Rasmussen KL, Laugesen CS, Ringholm L, Vestgaard M, Damm P, Mathiesen ER. Progression of diabetic retinopathy during pregnancy in women with type 2 diabetes. Diabetologia. 2010;53(6):1076-83. Epub 2010/03/13. doi: 10.1007/s00125-010-1697-9. PubMed PMID: 20225131.

39. Ringholm L, Pedersen-Bjergaard U, Thorsteinsson B, Boomsma F, Damm P, Mathiesen E. Higher levels of Atrial Natriuretic Peptide (ANP) are present in early pregnancy in type 1 diabetic women developing preeclampsia , September 2011, vol/is 54/. 2011:S485.

40. Ringholm L, Vestgaard M, Laugesen CS, Juul A, Damm P, Mathiesen ER. Pregnancy-induced increase in circulating IGF-I is associated with progression of diabetic retinopathy in women with type 1 diabetes. Growth Horm IGF Res. 2011;21(1):25-30. Epub 2011/01/08. doi: 10.1016/j.ghir.2010.12.001. PubMed PMID: 21212010.

41. Young EC, Pires ML, Marques LP, de Oliveira JE, Zajdenverg L. Effects of pregnancy on the onset and progression of diabetic nephropathy and of diabetic nephropathy on pregnancy outcomes. Diabetes Metab Syndr. 2011;5(3):137-42. Epub 2012/07/21. doi: 10.1016/j.dsx.2012.02.013. PubMed PMID: 22813566.

42. Abe Y, Mitani M, Fukazawa Y, Konno J, Makino Y, Matsuda Y. Pregnancy induced hypertension in women with pregestational diabetes mellitus. J Paediatr Child Health. 2012;48(117):1034-4810.

43. Bell R, Glinianaia SV, Tennant PW, Bilous RW, Rankin J. Peri-conception hyperglycaemia and nephropathy are associated with risk of congenital anomaly in women with pre-existing diabetes: a population-based cohort study. Diabetologia. 2012. Epub 2012/02/09. doi: 10.1007/s00125-012-2455-y. PubMed PMID: 22314812.

44. Themeli Y, Bajrami V, Zaimi K, Mustafaraj K, Lulo J, Peci E, et al. Diabetic nephropathy in pregnant women with type 1 diabetes mellitus. Giornale Italiano di Ostetricia e Ginecologia. 2012;34(1):323-7.

45. Damm JA, Asbjornsdottir B, Callesen NF, Mathiesen JM, Ringholm L, Pedersen BW, et al. Diabetic nephropathy and microalbuminuria in pregnant women with type 1 and type 2 diabetes: prevalence, antihypertensive strategy, and pregnancy outcome. Diabetes Care. 2013;36(11):3489-94. Epub 2013/09/07. doi: 10.2337/dc13-1031. PubMed PMID: 24009298; PubMed Central PMCID: PMCPMC3816914.

46. Patel N, Brackenridge A, Kanji A, Pasupathy D, Rajasingam D. Micro-vascular disease at booking in T1DM and associated risk of developing pre-eclampsia. Archives of Disease in Childhood: Fetal and Neonatal Edition. 2013;98:1359-2998.

47. Castiglioni MT, Valsecchi L, Cavoretto P, Pirola S, Di Piazza L, Maggio L, et al. The risk of preeclampsia beyond the first pregnancy among women with type 1 diabetes parity and preeclampsia in type 1 diabetes. Pregnancy Hypertens. 2014;4(1):34-40. Epub 2014/01/01. doi: 10.1016/j.preghy.2013.09.001. PubMed PMID: 26104252.

48. Herman M, Djelmis J, Ivanisevic M, Blajic J, Starcevic V. Pregnancy outcome of mothers with diabetic nephropathy. Journal of Maternal-Fetal and Neonatal Medicine. 2014;27:266-7.

49. Egan AM, McVicker L, Heerey A, Carmody L, Harney F, Dunne FP. Diabetic retinopathy in pregnancy: a population-based study of women with pregestational diabetes. J Diabetes Res. 2015;2015:310239. Epub 2015/05/07. doi: 10.1155/2015/310239. PubMed PMID: 25945354; PubMed Central PMCID: PMCPMC4402566.

50. Klemetti MM, Laivuori H, Tikkanen M, Nuutila M, Hiilesmaa V, Teramo K. White's classification and pregnancy outcome in women with type 1 diabetes: a population-based cohort study. Diabetologia. 2016;59(1):92-100. Epub 2015/10/18. doi: 10.1007/s00125-015-3787-1. PubMed PMID: 26474777.

51. Toda J, Kato S, Sanaka M, Kitano S. The effect of pregnancy on the progression of diabetic retinopathy. Jpn J Ophthalmol. 2016;60(6):454-8. Epub 2016/10/23. doi: 10.1007/s10384-016-0464-y. PubMed PMID: 27456842.

52. Durackova L, Kristufkova A, Korbel M. Pregnancy and neonatal outcomes in women with type 1 diabetes mellitus. Bratisl Med J. 2017;118(1):56-60.

53. Gutaj P, Zawiejska A, Mantaj U, Ożegowska E. Determinants of preeclampsia in women with type 1 diabetes. Acta Diabetol. 2017;54:1115-21.

54. Norgaard SK, Vestgaard MJ, Jorgensen IL, Asbjornsdottir B, Ringholm L, McIntyre HD, et al. Diastolic blood pressure is a potentially modifiable risk factor for preeclampsia in women with pre-existing diabetes. Diabetes Res Clin Pract. 2018;138:229-37. Epub 2018/02/24. doi: 10.1016/j.diabres.2018.02.014. PubMed PMID: 29475019.

55. Samii L, Kallas-Koeman M, Donovan LE, Lodha A, Crawford S, Butalia S. The association between vascular complications during pregnancy in women with Type 1 diabetes and congenital malformations. Diabet Med. 2019;36(2):237-42. Epub 2018/12/01. doi: 10.1111/dme.13872. PubMed PMID: 30499197.

56. Bourry J, Courteville H, Ramdane N, Drumez E, Duhamel A, Subtil D, et al. Progression of Diabetic Retinopathy and Predictors of Its Development and Progression During Pregnancy in Patients With Type 1 Diabetes: A Report of 499 Pregnancies. Diabetes Care. 2021;44(1):181-7. Epub 2020/11/13. doi: 10.2337/dc20-0904. PubMed PMID: 33177172.
