# Supplementary material for: Adverse pregnancy outcomes in women with diabetes-related microvascular disease and risks of disease progression in pregnancy: A systematic review and meta-analysis
Source: PLoS Med. 2021 Nov 22;18(11):e1003856. doi: 10.1371/journal.pmed.1003856 (PMC8654151; doi:10.1371/journal.pmed.1003856)
Supplement: S6 Appendix — Table A: Maternal and perinatal outcomes in women with type 1 diabetes and vasculopathy—A sensitivity analysis. Table B: Maternal and perinatal outcomes in women with diabetes and vasculopathy—A sensitivity analysis excluding papers with high risk of bias. Table C: Risk factors for disease progression (retinopathy)—Sensitivity analyses. (DOCX) [file pmed.1003856.s006.docx]

**S6 Appendix – Results of sensitivity analyses**

**Table A: Maternal and perinatal outcomes in women with type 1 diabetes and vasculopathy – a sensitivity analysis**

| Risk factor | Outcome | | No. of studies | No of women | OR (95% CI) | Heterogeneity I^2^ (%) | P- Value |
| --- | --- | --- | --- | --- | --- | --- | --- |
| Nephropathy | Caesarean section | | 3 | 1485 | 4.03 (0.97-16.80) | 82% | 0.06 |
|  | Pre-eclampsia | | 8 | 2009 | 12.40 (5.75- 26.77) | 73% | <0.01 |
|  | Pregnancy induced hypertension | | 6 | 2245 | 2.69 (1.255-5.76) | 72% | 0.01 |
|  | Congenital anomaly | | 3 | 502 | 2.52 (0.97-6.57) | 0% | 0.06 |
|  | Large for gestational age | | 3 | 325 | 0.34(0.17-0.67) | 0% | <0.01 |
|  | Small for gestational age | | 0 | - | - | - | - |
|  | Premature birth < 37/40 | | 5 | 1713 | 5.02(3.10-8.14) | 23% | <0.01 |
|  | Premature birth < 34/40 | | 4 | 619 | 6.81(2.31-20.06) | 44% | <0.01 |
|  | Neonatal hypoglycaemia | | 2 | 1135 | 1.38(0.60- 3.18) | 47% | 0.45 |
|  | Neonatal unit admission | | 1 | 1094 | 2.56 (1.66-3.93) | - | <0.01 |
|  | Perinatal deaths | | 6 | 2198 | 2.26 (1.07-4.75) | 0% | 0.03 |
|  | |  |  |  |  |  | |
| Retinopathy | Caesarean section | | 2 | 144 | 7.37 (0.12-458.28) | 86% | 0.34 |
|  | Pre-eclampsia | | 7 | 2822 | 1.97 (1.44-2.71) | 28% | <0.01 |
|  | Pregnancy induced hypertension | | 5 | 2803 | 1.32 (1.00-1.75) | 0% | 0.05 |
|  | Congenital anomaly | | 1 | 60 | 18.73 (0.96-366.50) | - | 0.05 |
|  | Large for gestational age | | 1 | 16 | 1.00 (0.10-9.61) | - | 1.00 |
|  | Premature birth < 37/40 | | 1 | 1094 | 1.38 (0.94-2.01) | - | 0.10 |
|  | Neonatal hypoglycaemia | | 1 | 1094 | 1.01 (0.69-1.47) | - | 0.10 |
|  | |  |  |  |  |  | |
| Nephropathy & Retinopathy | Caesarean section | | 3 | 352 | 5.74 (1.89-17.40) | 42% | <0.01 |
|  | Pre-eclampsia | | 8 | 1232 | 5.32(3.52- 8.05) | 12% | <0.01 |
|  | Pregnancy induced hypertension | | 1 | 165 | 2.33 (0.10-51.95) | 87% | 0.59 |
|  | Congenital anomaly | | 2 | 307 | 1.23 (0.70-2.15) | 0% | 0.47 |
|  | Large for gestational age | | 4 | 696 | 1.10 (0.80-1.52) | 0% | 0.56 |
|  | Premature birth < 37/40 | | 6 | 1902 | 2.20 (1.75-2.77) | 0% | <0.01 |
|  | Neonatal hypoglycaemia | | 4 | 659 | 1.09 (0.56-2.14) | 56% | 0.80 |
|  | Neonatal unit admission | | 2 | 1291 | 1.21 (0.91-1.63) | 0% | 0.19 |
|  | Perinatal deaths | | 4 | 1617 | 0.91 (0.35-2.34) | 20% | 0.84 |

**Table B: Maternal and perinatal outcomes in women with diabetes and vasculopathy – a sensitivity analysis excluding papers with high risk of bias.**

| Risk factor | Outcome | | No. of studies | No. of women | OR (95% CI) | Heterogeneity I^2^ (%) | P- Value |
| --- | --- | --- | --- | --- | --- | --- | --- |
| Nephropathy | Caesarean section | | 4 | 1631 | 3.79 (1.42-10.15) | 73% | 0.01 |
|  | Pre-eclampsia | | 9 | 2730 | 8.92 (5.27-15.09) | 65% | <0.01 |
|  | Pregnancy induced hypertension | | 6 | 2245 | 2.69 (1.26-5.76) | 72% | 0.01 |
|  | Congenital anomaly | | 5 | 2436 | 2.62 (1.51-4.53) | 0% | <0.01 |
|  | Large for gestational age | | 2 | 299 | 0.37 (0.18-0.77) | 0% | 0.01 |
|  | Small for gestational age | | 4 | 583 | 25.75 (10.51-63.08) | 0% | <0.01 |
|  | Premature birth < 37/40 | | 6 | 2024 | 4.39 (3.16-6.10) | 8% | <0.01 |
|  | Premature birth < 34/40 | | 6 | 1076 | 6.10 (2.89-12.85) | 49% | <0.01 |
|  | Perinatal deaths | | 5 | 1859 | 2.46 (1.14-5.32) | 0% | 0.02 |
|  | |  | | | | | |
| Retinopathy | Caesarean section | | 2 | 144 | 7.37 (0.12-458.28) | 86% | 0.34 |
|  | Pre-eclampsia | | 6 | 2770 | 2.22 (1.47- 3.35) | 55% | <0.01 |
|  | Pregnancy induced hypertension | | 4 | 2341 | 1.21 (0.89-1.65) | 0% | 0.23 |
|  | Congenital anomaly | | 2 | 1754 | 3.30 (0.27-39.83) | 67% | 0.35 |
|  | Large for gestational age | | 1 | 482 | 1.97 (1.00-3.90) | - | 0.05 |
|  | Premature birth < 37/40 | | 2 | 1576 | 1.61 (1.06-2.43) | 32% | 0.02 |
|  | Perinatal deaths | | 1 | 1094 | 0.26 (0.04-1.94) | - | 0.19 |
|  |  | |  |  |  |  |  |
| Nephropathy & Retinopathy | Caesarean section | | 3 | 716 | 6.03 (2.63-13.79) | 34% | <0.01 |
|  | Pre-eclampsia | | 6 | 977 | 5.04 (3.07-8.30) | 10% | <0.01 |
|  | Pregnancy induced hypertension | | 2 | 302 | 0.95 (0.16-5.73) | 20% | 0.96 |
|  | Congenital anomaly | | 1 | 189 | 1.15 (0.52- 2.53) | - | 0.74 |
|  | Large for gestational age | | 3 | 578 | 1.02 (0.72-1.46) | 0% | 0.90 |
|  | Small for gestational age | | 3 | 575 | 2.98 (1.02-8.75) | 0% | 0.05 |
|  | Premature birth < 37/40 | | 5 | 2118 | 2.43 (1.94-3.04) | 0% | <0.01 |
|  | Premature birth < 34/40 | | 2 | 245 | 5.32 (1.33-21.38) | 65% | 0.02 |
|  | Perinatal deaths | | 3 | 1499 | 1.31 (0.31-5.61) | 46% | 0.72 |

**Table C: Risk factors for disease progression (retinopathy) – sensitivity analyses**

| Sensitivity analysis | Risk factor | | No. of studies | No. of women | OR (95% CI) | Heterogeneity I^2^ (%) | P- Value |
| --- | --- | --- | --- | --- | --- | --- | --- |
| Women with type 1 diabetes only | Background/pre-proliferative retinopathy | | 3 | 331 | 2.68 (1.58-4.54) | 0.0% | <0.01 |
|  | Proliferative retinopathy | | 6 | 410 | 2.04 (0.89-4.67) | 41.5% | 0.09 |
|  | Macular oedema | | 1 | 88 | 1.61 (0.43-5.98) | N/A | 0.48 |
|  | Previous photocoagulation | | 2 | 553 | 0.37 (0.09-1.50) | 15.7% | 0.16 |
|  | Any retinopathy | | 11 | 1,489 | 2.27 (1.14-4.55) | 80.5% | 0.02 |
|  | Nephropathy | | 4 | 849 | 1.68 (1.05-2.69) | 0.0% | 0.03 |
|  | White ethnicity | | 1 | 205 | 2.06 (0.67-6.33) | N/A | 0.21 |
|  | Nulliparity | | 4 | 980 | 1.75 (1.28-2.40) | 0.0% | <0.01 |
|  | Smoking | | 5 | 456 | 2.31 (1.25-4.27) | 0.0% | <0.01 |
|  | |  | | | | | |
| Excluding studies with a high risk of bias | Background/pre-proliferative retinopathy | | 1 | 140 | 2.91 (1.30-6.50) | N/A | 0.01 |
|  | Proliferative retinopathy | | 2 | 173 | 1.44 (0.64-3.24) | 0.0% | 0.38 |
|  | Macular oedema | | 1 | 88 | 1.61 (0..43-5.98) | N/A | 0.48 |
|  | Previous photocoagulation | | 1 | 499 | 0.23 (0.06-0.99) | N/A | 0.049 |
|  | Any retinopathy | | 6 | 1,062 | 2.00 (0.85-4.71) | 82.9% | 0.11 |
|  | Nephropathy | | 4 | 849 | 1.68 (1.05-2.69) | 0.0% | 0.03 |
|  | White ethnicity | | 1 | 205 | 2.06 (0.67-6.32) | N/A | 0.21 |
|  | Nulliparity | | 4 | 980 | 1.75 (1.28-2.40) | 0.0% | <0.01 |
|  | Smoking | | 2 | 185 | 1.13 (0.17-7.60) | 63.2% | 0.90 |
