# Supplementary material for: Adverse pregnancy outcomes in women with diabetes-related microvascular disease and risks of disease progression in pregnancy: A systematic review and meta-analysis
Source: PLoS Med. 2021 Nov 22;18(11):e1003856. doi: 10.1371/journal.pmed.1003856 (PMC8654151; doi:10.1371/journal.pmed.1003856)
Supplement: S7 Appendix — (DOCX) [file pmed.1003856.s007.docx]

**S7 Appendix – Funnel plots for publication bias assessments**

**Progression of retinopathy: P = 0.003**

**Pre-eclampsia: P = 0.001**
