# Supplementary material for: Adverse pregnancy outcomes in women with diabetes-related microvascular disease and risks of disease progression in pregnancy: A systematic review and meta-analysis
Source: PLoS Med. 2021 Nov 22;18(11):e1003856. doi: 10.1371/journal.pmed.1003856 (PMC8654151; doi:10.1371/journal.pmed.1003856)
Supplement: Attachment — Submitted filename: Dr Sophie Relph-PloS medicine review 26 June 2021.docx [file pmed.1003856.s010.docx]

PMEDICINE-D-21-02305R1. Drs. Sophie Relph *et al.* 2021. "Adverse pregnancy outcomes in women with diabetes-related microvascular disease and risks of disease progression in pregnancy: A systematic review and meta-analysis."

**Summary of the research and reviewer's overall impression.**

**Summary:** Focusing on pregnancy outcomes in women with preexisting diabetes and microvascular disease, the authors made a critical, prospective, concise, and incisive review of the literature and performed a meta-analysis of banked data from 12,320 pregnancies in 55 studies spanning almost 30 years. The great majority of those studies (~87%) were conducted in Europe and North America. Two independent reviewers screened papers for inclusion, and a third independent reviewer adjudicated conflicts. The 55 reports were winnowed from 2,811 citations initially selected. Results are expressed as odds ratios (OR).

Considering pregnancies with both preexisting maternal nephropathy and diabetes, compared to those with diabetes only:

- Mothers were at greatly increased OR risk of both early (<34 weeks) and any preterm delivery.
- Offspring were at increased OR risk of congenital abnormalities, being small for gestational age (SGA), having needed admission to the NICU, and perinatal death.

Similarly, preexisting diabetic retinopathy in moms was associated with increased OR risk of preeclampsia and preterm delivery.

Morbidity of retinopathy was elevated in moms with nulliparity*, smoking, preexisting proliferative disease, and prior duration of diabetes.

Doctor Relph and her colleagues conclude that "In pregnant women with diabetes, presence of nephropathy and/or retinopathy further increase[s] the risks of maternal complications."

**Reviewer's summary statement:** This manuscript provides a helpful and timely primer on a growing and difficult health challenge, worldwide. It is an enjoyable read. A complex primary literature is artfully distilled into understandable queries into the problem at hand. Quality measures are explicit (lines 146-154; Figure 2). This new meta-analysis, the largest such study to date, provides clear, evidence-based guidance that obstetric and endocrine clinicians need to intervene early and aggressively in these at-risk women, for the sake of both the moms and their children. The authors’ synthesis succeeds in being quantitative, rather than merely narrative. This reviewer predicts that the present work will be highly cited, and that it will help to inform favorable changes in medical policy and practice.

**Disclosure regarding this reviewer's qualifications:** This basic-science investigator studies metabolism of the major human metabolic diseases, including gestational diabetes. This reviewer is neither a clinician nor a statistician.

**Evidence and examples. Specific questions and concerns:**

This manuscript rightly focuses on short-term outcomes in mother and child. In the few longer-term studies that have been published, what are the later-in-life consequences of women becoming pregnant while they are both diabetic and afflicted with microvascular disease?

Definitions of most patient characteristics and medical conditions are explicitly given in the text (e.g., lines 120-143, 181-198). Multicenter, observational data considered here span more than 30 years of medical practice, and the clinical definitions of several concepts evolved over the decades concerned. *For example, use of the adjective "nulliparous" and related terms here (abstract, last sentence of Findings; Table 2; Leperco entry in page 9 of the appendices; and in manuscript lines 62, 290, 330, 399, and 457) might confuse some readers because its definition has varied across time and space—is it meant to signify "mothers who for the first time carried pregnancies to term" here?

Institutional review boards and informed consent by human subjects: Pursuant to the *PLoS Medicine*’s guidelines (pages 4 and 6, left sidebars), could the authors please provide an appendicular table that summarizes the names of the Institutional Review Boards with authority over the 55 observational studies included here, and noting whether or not the original, on-site investigators confirmed that the subjects’ consent was obtained, consistent with the Declaration of Helsinki, or some similar international standard?

Heavy weighting toward Euro-North American studies (lines 182-183) should be noted briefly in the abstract. Outcomes could well be different in, say, South-Asian and Chinese populations.

Association scores between diabetic retinopathy and Caesarean birth are strikingly broad (Figure 4, fourth line down). Could the authors please comment on this briefly in the text? Does this relate to how medical practices differ among the geographic regions under consideration?

Authors acknowledge (*e.g.,* lines 348-351) that limitations of many of the 55 constitutive publications do not allow a thorough stratification of pregnancy outcomes in Type 1 *versus* Type 2 diabetes, which would have been helpful, and should be addressed in future studies.

**Other point / minor copy edit.**

Meaning of the phrase “at booking” (lines 304 and 427) might not be readily apparent to some readers whose first language is not British English.
