# Supplementary material for: Adverse pregnancy outcomes in women with diabetes-related microvascular disease and risks of disease progression in pregnancy: A systematic review and meta-analysis
Source: PLoS Med. 2021 Nov 22;18(11):e1003856. doi: 10.1371/journal.pmed.1003856 (PMC8654151; doi:10.1371/journal.pmed.1003856)
Supplement: Attachment — Submitted filename: Response to editors.docx [file pmed.1003856.s012.docx]

**Adverse pregnancy outcomes in women with diabetes-related microvascular disease and risks of disease progression in pregnancy: a systematic review and meta-analysis**

**(PMEDICINE-D-21-02305R1)**

**Standard requests**

1. We ask every co-author listed on the manuscript to fill in a contributing author statement. If any of the co-authors have not filled in the statement, we will remind them to do so when the paper is revised. If all statements are not completed in a timely fashion this could hold up the re-review process. Should there be a problem getting one of your co-authors to fill in a statement we will be in contact. YOU MUST NOT ADD OR REMOVE AUTHORS UNLESS YOU HAVE ALERTED THE EDITOR HANDLING THE MANUSCRIPT TO THE CHANGE AND THEY SPECIFICALLY HAVE AGREED TO IT.

We have provided an ICJME Form for Disclosure of Potential Conflicts of Interest for each author via the submission system. Please let us know if you require any additional statements.

2. Please ensure that the paper adheres to the PLOS Data Availability Policy, which requires that all data underlying the study's findings be provided in a repository or as Supporting Information. For data residing with a third party, authors are required to provide instructions with contact information for obtaining the data. PLOS journals do not allow statements supported by "data not shown" or "unpublished results." For such statements, authors must provide supporting data or cite public sources that include it.

All data included in meta-analyses have now been included as supporting information (S8 and S9 Appendix).

3. To enhance the reproducibility of your results, we recommend that you deposit your laboratory protocols in [protocols.io](http://protocols.io), where a protocol can be assigned its own identifier (DOI) such that it can be cited independently in the future. Additionally, PLOS ONE offers an option to publish peer-reviewed clinical study protocols.

As has already been referenced in the manuscript (line 137-138), our analysis protocol was published on the PROSPERO website (CRD42017076647).

4. Please review your reference list to ensure that it is complete and correct. If you have cited papers that have been retracted, please include the rationale for doing so in the manuscript text, or remove these references and replace them with relevant current references. Any changes to the reference list should be mentioned in the rebuttal letter that accompanies your revised manuscript.

We believe that the reference list is correct (following changes made as requested below).

**Editors comments**

5. Please remove “Funding” from the abstract.

This has been removed.

6. Please remove the “Data availability statement” and “Declaration of interest” from the end of the main text. In the event of publication, this information will be published as metadata based on your responses to the submission form.

We have removed both statements.

7. Please do not report P<0.01; report as P < 0.001.

We have checked these p values and changed p<0.01 to p<0.001 in all cases (as appropriate).

8. Please place in-text reference call outs prior to punctuation noting the space between the last word and call out. For example, “…countries [1,2].”

We have edited the reference call outs as requested.

9. Please ensure that journal name abbreviations consistently match those found in the National Center for Biotechnology Information (NCBI) databases.

We believe that these are all correct.

10. Please include access date for ref #10, 12 & 76.

We have added the access date for refs #10 and #76. Ref #12 already has an access date provided.

11. For additional emphasis, please pay special attention to reviewer #2’s comments.

Responses to reviewer #2’s comments provided below.

**Reviewer #1:**

12. The authors have addressed my concerns and I now recommend publication.

**Reviewer #2:**

13. The combination of T1 and Y2 diabetes in this Review is too important to omit from the Abstract. The authors state the omission is due to space limits. However, making room is simple. For example, if they insert 'Type 1 or 2' in line 47 of their Abstract's text in front of 'diabetic microvascular disease' then in line 53, remove 'of these were' in front of 'Europe', and remove the 'and' after 'Europe', replacing it with a comma, the word count remains the same as now.

We previously misunderstood reviewer #2’s request. We have made this minor edit as suggested.

14. This reviewer does not accept that confidence intervals should be to 2 decimal points when throughout, the number of events are frequently small, and types of diabetes are combined. As previously in the reviewer's point, now no.23, with respect it is false precision and not accuracy to quote such detail. The editors can advise.

We have referred to existing examples of published meta-analyses in PLOS Medicine during 2020-2021. Of the top 10 search results for meta-analysis during this time frame that reported odds ratios (ORs), all used two decimal points. We will be guided by the suggestions of the editorial team prior to making any changes.

15. Finally, and surely, the job of a systematic review when risks such as these are so high is not just to say so but to indicate where further work could usefully perhaps reduce them. While blood pressure (BP) may be important, it is obviously imprecise especially when taken in a clinic. More modern techniques, which need not be complex and appear to be more stable, are available, as cited previously. Testing these out could offer more than badly taken, routine BP, as could home BP measures, or occasionally 12 or 24 hour values. Similarly simple retinal or renal tests, advised and perhaps tested by collaborating specialists they suggest to involve, might be tried. Commenting that they are not worth mentioning because they are not currently routine would mean nothing novel would ever get introduced.

We have added further detail to the section ‘Recommendations for research’. We have added the phrase ‘*or preferred method for the assessment of renal function decline*’ (line 502/503) to the existing sentence ‘*We need consensus on criteria for deterioration of diabetic nephropathy in pregnancy to identify the proportion of women with worsening disease and the risk factors for disease progression*.’ We have also added a sentence about improving the assessment of blood pressure control: ‘*Techniques worthy of further assessment for this purpose include continuous measurement of blood pressure over 12- or 24-hour periods, or provision of validated monitors for home monitoring*.’ (line 506-508).

**Reviewer #3:**

16. Doctors Relph and colleagues made thoughtful responses to the editor's and reviewers' comments. Reassuringly, their updated meta-analysis supported their earlier conclusions. In-depth queries regarding statistics and the authors' responses thereto are largely beyond this reviewer's competence and experience.
17. Regarding point 35, this reviewer understands, but respectfully disagrees that an on-line compendium of IRB and similar ethical - review - board approvals is not essential here.

As per the response to point 14 above, we have referred to the top 10 meta-analyses reporting ORs in the PLOS Medicine online search for 2020-2021 publications. Only one of these ten meta-analyses provided an online compendium of institutional review boards for all included cohorts. In this case, it was an individual participant data meta-analysis for which we agree that it was necessary to present IRB details. None of the remaining 9 meta-analyses reported on this. We will be guided by the suggestions of the editorial team on this.
