# Supplementary material for: Adverse pregnancy outcomes in women with diabetes-related microvascular disease and risks of disease progression in pregnancy: A systematic review and meta-analysis
Source: PLoS Med. 2021 Nov 22;18(11):e1003856. doi: 10.1371/journal.pmed.1003856 (PMC8654151; doi:10.1371/journal.pmed.1003856)
Supplement: Attachment — Submitted filename: Response to reviewer comments.docx [file pmed.1003856.s011.docx]

**Adverse pregnancy outcomes in women with diabetes-related microvascular disease and risks of disease progression in pregnancy: a systematic review and meta-analysis**

**(PMEDICINE-D-21-02305R1)**

**Editors comments**

1. Please report your abstract according to PRISMA for abstracts, following the PLOS Medicine abstract structure,

2. Please structure your abstract using the PLOS Medicine headings (Background, Methods and Findings, Conclusions).

3. Please combine the [Abstract] Methods and Findings sections into one section, “Methods and findings”.

4. Please revise the [Abstract] subheading “Interpretation” to “Conclusions.”

As requested, we have restricted the abstract with reference to the PLOS Medicine required headings and the PRISMA for abstracts checklist (pg 3-4).

2. Please ensure that all numbers presented in the abstract are present and identical to numbers presented in the main manuscript text.

We have checked numbers throughout the abstract and main text for accuracy and made corrections where necessary.

3. Please include p values in addition to 95% Cis [in the abstract]

As requested, we have added p values to all quoted statistics in the abstract (pg 3-4).

4. In the last sentence of the Abstract Methods and Findings section, please describe the main limitation(s) of the study's methodology.

We have now added the following sentence to the methods and findings section of the abstract: ‘*The main limitations of this analysis are the heterogeneity of definition of retinopathy and nephropathy and of inclusion of women both with type 1 and type 2 diabetes.’* (page 4, line 66)

5. [Abstract] Please address the study implications, stating what is new, without overreaching what can be concluded from the data; the phrase "In this study, we observed ..." may be useful.

We have revised the last sentence of the abstract to read: *‘In pregnant women with diabetes, presence of nephropathy and/or retinopathy appear to further increase the risks of maternal complications.’ (page 4, line 70)*

6. Author summary - At this stage, we ask that you reformat your non-technical Author Summary. The Author Summary should immediately follow the Abstract in your revised manuscript. This text is subject to editorial change and should be distinct from the scientific abstract. The summary should be accessible to a wide audience that includes both scientists and non-scientists. Please see our author guidelines for more information.

We have added this new section on pages 5-6 following the abstract.

7. Please update your search to the present time.

We have updated the search to July 2021. During this process, we identified 174 new titles but following title/abstract review, only one paper was relevant to this meta-analysis. This paper added data on risk factors associated with progressive diabetic retinopathy disease. We have therefore re-run this analysis and revised all relevant statistics and tables in the submission. The overall findings have not changed. We have also updated Figure 1 by adding the newly identified studies.

8. Please evaluate evidence of publication bias.

We have included an evaluation of publication bias for all results for which there were at least 10 studies contributing. The methods are reported in line 205, page 11, results on line 371, pg 18 and comment in the discussion (line 463, page 22).

9. PRISMA checklist - when completing the checklist, please use section and paragraph numbers, rather than page numbers.

The PRISMA checklist has been resubmitted using section and paragraph numbers.

10. In the Methods and Results section - Please provide 95% CIs and p values for estimates in the main text and tables

We have added p values to all estimates in the main text and also to Tables 1 and 2.

11. Please name Fig 1, “PRISMA flow chart…”

Figure 1 has been renamed as requested.

12. In discussion section, please move the “strengths and limitations” paragraph further down and place before “Implications for clinical practice”.

This section has been moved as requested and is now reported in paragraphs 6-7 of the discussion section.

13. Please use bars and whiskers in the forest plots (Figure 3 & 4), and indicate in the figure caption the meaning of the bars and whiskers.

Figures 3 and 4 show summary estimates only and are not estimates of the individual studies. We have therefore provided a diamond rather than box/whisker, as is standard for summary estimates. We have now added a description of the meaning of the diamond symbol in the figure legends: ‘**The diamond symbols represent the point estimate at the central point and the 95% confidence interval at the left (lower interval) and right (upper interval) extremes*.’

If the Editors would also like additional figures that demonstrate the forest plot with each additional study for all outcomes, these can be provided. But we estimate that there will be 30 forest plots, and can be included in supplementary material.

14. Please replace "Caucasian" with "white" throughout the paper.

We have replaced the single use of the word ‘Caucasian’ with ‘White’ as requested on Table 2.

15. Please ensure standard reference formatting:
a)	Please use the "Vancouver" style for reference formatting, and see our website for other reference guidelines 
b)	Please use the PLOS Medicine style reference call outs throughout the text, noting the absence of spaces within the square brackets, e.g., "... between 1980 and 2014 [1,2]."
c)	Please ensure that weblinks are current and include date of access.

Reference citations have been edited throughout the text to remove spaces from the previous formatting. All weblinks are current and include the date of access where appropriate.

**Reviewer 1**

16. I confine my remarks to statistical aspects of this paper. The general approach is fine but I do have a couple issues to resolve before i can recommend publication.

We thank reviewer 1 for their time and statistical expertise in reviewing this paper. We are pleased to hear that the reviewer believes that the manuscript be recommended for publication once the points outlined below have been resolved.

17. General: When I^2 is high, it's good to try to figure out why.

We have added an additional comment in the Discussion section *(page 22, line 461)* regarding the potential causes of heterogeneity as follows.

*‘* Furthermore, the variations in the definitions of the populations, retinopathy and nephropathy may have contributed to the high heterogeneity observed in some findings.’

18. p. 7 line 128-132  Why these cutoffs? This means that, even if the sample is typical, 20% will be called either small or large. It would be better if there were substantive reasons and values for weight that mark abnormality or difficulty.

The definitions for small and large for gestational age babies are consistent with those in common clinical practice, as recommended by international guidelines on this topic. The included studies also used the same definitions, as these babies are known to be at higher risk of complications.

19. Fig 2  "Number of studies"  - this seems like it is a % of studies, not an N. A parallel box plot seems better suited for these data. Don't divide quality into 3 categories, use the actual quality score

In response to reviewer 1’s suggestions, we have revised Figure 2 as requested. The x-axis now represents the percentage of studies which fall into each quality score category.

We have also added the actual quality score, alongside the categorical result, for each study to the table in Supplementary Material 5.

20. Fig 3 and 4
      .01 on one side corresponds to 100 on the other, so those two should be marked
       it would be good to mark more than two points, e.g. have .01, .05, .1, ,5, 1, 2, 5, 10, 20, 100  or something like thatr
       Maybe put the ORs and p values in a table, rather than here, to allow more room for the graph
       Consider using landscape view for the same reason

The current x-axis points are placed on the upper and lower limits of all points on the graph. Additional tick points could be added but we are concerned that they would lead to a loss of clarity in the x-scale. Both the point estimates and upper and lower 95% CI limits for each diamond/outcome are reported in the figure to ensure the reader can easily look up exactly where these fall – and does not need to try to interpret the x-axis. We believe it is preferable to include these here, rather than requiring a reader to refer to a separate table which would partly duplicate the information.

However, if the Editors agree that it would be better to remove the information from the graph and instead report in a table, or to present the graph in landscape view, then we would be happy to rearrange the data.

**Reviewer 2**

21. As the authors say, this appears to be the only major quantitative as opposed to narrative Syst. Rvw available of this growing issue. It is therefore important work. The results are dramatic if with wide confidence intervals (CI) reflecting the relatively small nos of studies & also events. However, the global importance both clinically & to Public Health are clear; obesity -T2 Diabetes (still rather under-represented, as here, it seems)- complications earlier in life and now pregnancy continue as a major threat. I have no major methodological points but some minor ones.

We are grateful for reviewer 2’s time and completely agree about the clinical importance of this meta-analysis on a subject that has not previously been summarised in this way. Pre-existing diabetes is an increasing co-morbidity amongst pregnant women, largely driven by the obesity epidemic. We therefore consider this work to be extremely timely and important.

22. They review papers from 1990-2020; were there hints or overt signs of time trends as identifying these complications pre- or in pregnancy improved?

Whilst we agree that this is important, we do not think that we will have sufficient sample size to provide a definitive finding on the differential association between diabetes and maternal or perinatal adverse outcomes by year of publication.

We have previously addressed the effect of time as a limitation in the discussion on lines 458 (pg 22) as follows ‘*We only included studies published since 1990, but it is possible that some of the outcomes could be influenced by the variations in clinical practice over time and between institutions*.’ As we do not consider this to be the main limitation of the analysis, we have not also included this in the abstract.

23. Numbers of events in these high Odds Ratios, as with the nos. of studies with each complication (nephropathy/ retinopathy etc), were relatively SMALL, obviously contributing to the wide CIs as above - they can say so.

We have added a line in the discussion of limitations to this effect. ‘*There were very few events for some of the reported outcomes studied. This is reflected in the imprecision of the point estimates.*’ *(page 22, line 460)*

24. These CI should be only 1 decimal point throughout the text - false precision beyond.

We have chosen to include two decimal points to report results in the text because we think that this aids accuracy. We believe that the degree of precision is demonstrated by the breadth confidence intervals.

However, if the Editors request so, we would be happy to change this prior to publication.

25. Despite point [22], under 'Interpretation' here, they are entitled to say '..further greatly increase…..risks..';

We have refrained from using the word ‘greatly’, a subjective term.

26. There is no mention here in the Abstract of why they consider 'diabetes' as a whole = that is the no of studies including Type 2DM were still few.

Type 1 and type 2 diabetes both predispose to the same maternal and perinatal complications, we have described this in the introductory section of the main manuscript. Due to limitations of word count, we have refrained from including this in the abstract also.

27. Results:	Again include here the N for Type 1 vs/+ Type 2 studies;

This is already reported on lines 224, pg 11.

‘*Twelve studies included women with both type 1 and type 2 diabetes, 29 only type 1 diabetes, six only insulin-dependent diabetes, one type 2 diabetes and seven unspecified diabetes type’*.

28. Discussion:	2nd line (no.326) - they could add  'even' as '…at EVEN greater risk..', given that the additive risk of pre-eclampsia is already much higher than without DM.

We have added the word ‘even’ as suggested to the line (now line 379).

29. [Discussion] They highlight the current confusion over how blood pressure features due to highly variable classification etc.; they might make rationalising this a central recommendation - AND that other blood vessel measures are available for analysing the excess risk (aortic PWV/ carotid / basic cardiac measures - all surely ready for clinical testing as risk indicators (see  [doi.org/10.1002/uog.19021](http://doi.org/10.1002/uog.19021)).

We agree with the reviewer about the potential of these techniques. We haven’t included the suggested measures because these are not routinely conducted clinical tests.

We have now added a sentence in the Discussion section under research recommendations: ‘*There also needs to be a standardised method of assessing both glycaemic and blood pressure control throughout pregnancy, to determine what effect these have on progression of microvascular complications’*. *(page 24, lines 502)*

30. Refs: Their ref. 83 presumably is 'in press'.

We have updated the details of this reference which has now been published.

31. Figure 3: the lower risk for nephropathic mothers of LGA infants (OR 0.33 there) contradicts the similar line in Table 2 (between lines 279-280) with an OR of 1.1, admittedly including retinopathic mothers also, with larger Ns. Is this discrepancy correct? If so, it emphasises the instability of the ORs generally - which they might point out themselves (? In the Abstract & Discussion/ Limitations).

We have checked these figures which are correct. With nephropathy only, there is a consistent observation of reduced fetal growth (increased risk of SGA and decreased risk of LGA). When we include retinopathy as a risk factor in addition to/instead of nephropathy, the association with reduced fetal growth is lost – this is also seen in Figure 4. This demonstrates the unique effect that nephropathy has on fetal growth, which is not also seen with retinopathy.

**Reviewer 3**

32. Reviewer's summary statement:  This manuscript provides a helpful and timely primer on a growing and difficult health challenge, worldwide.  It is an enjoyable read.  A complex primary literature is artfully distilled into understandable queries into the problem at hand.  Quality measures are explicit (lines 146-154; Figure 2).  This new meta-analysis, the largest such study to date, provides clear, evidence-based guidance that obstetric and endocrine clinicians need to intervene early and aggressively in these at-risk women, for the sake of both the moms and their children.  The authors' synthesis succeeds in being quantitative, rather than merely narrative.  This reviewer predicts that the present work will be highly cited, and that it will help to inform favourable changes in medical policy and practice.

We thank reviewer 3 for their time and insight with regards to our manuscript. We are pleased to see that reviewer 3 agrees that this manuscript is likely to be highly cited because this is a helpful and timely submission of a growing health challenge.

33. This manuscript rightly focuses on short-term outcomes in mother and child.  In the few longer-term studies that have been published, what are the later-in-life consequences of women becoming pregnant while they are both diabetic and afflicted with microvascular disease?

We have already commented on the need for review of studies which present the longer-term risks of pregnancy with diabetic microvascular complications, and have now revised in the Discussion section as follows

‘*We need data on long-term outcomes beyond pregnancy for women with diabetic microvascular complications and their babies, to obtain a comprehensive overview of the risks. This information is critical to predict the risk of disease progression during pregnancy and postnatally*.’ *(page 24, line 508)*

34. Definitions of most patient characteristics and medical conditions are explicitly given in the text (e.g., lines 120-143, 181-198).  Multicenter, observational data considered here span more than 30 years of medical practice, and the clinical definitions of several concepts evolved over the decades concerned.  *For example, use of the adjective "nulliparous" and related terms here (abstract, last sentence of Findings; Table 2; Leperco entry in page 9 of the appendices; and in manuscript lines 62, 290, 330, 399, and 457) might confuse some readers because its definition has varied across time and space—is it meant to signify "mothers who for the first time carried pregnancies to term" here?

Nulliparity is an accepted terminology used by clinicians, researchers and guideline bodies. It refers to a woman who is carrying her first pregnancy beyond the gestation of viability. The reviewer is correct in noting that the gestation of expected viability has changed during the thirty years of study, and also may differ slightly by country. We have accepted the definitions used by the original study authors in all cases.

35. Institutional review boards and informed consent by human subjects:  Pursuant to the PLoS Medicine's guidelines (pages 4 and 6, left sidebars), could the authors please provide an appendicular table that summarizes the names of the Institutional Review Boards with authority over the 55 observational studies included here, and noting whether or not the original, on-site investigators confirmed that the subjects' consent was obtained, consistent with the Declaration of Helsinki, or some similar international standard?

All primary studies were published in journals and have been accepted. We expect the primary journals to have ensured that the research was conducted in line with ethics protocols at the time of publication.

36. Heavy weighting toward Euro-North American studies (lines 182-183) should be noted briefly in the abstract.  Outcomes could well be different in, say, South-Asian and Chinese populations.

We have included a line in the abstract to report the study characteristics: ‘*We included 56 cohort studies involving 12,819 pregnant women with diabetes; 40 of these studies were from Europe and 9 from North America*.’ *(page 3, lines 53)*

37. Association scores between diabetic retinopathy and Caesarean birth are strikingly broad (Figure 4, fourth line down).  Could the authors please comment on this briefly in the text?  Does this relate to how medical practices differ among the geographic regions under consideration?

Please see our responses to comments 17 and 23.

38. Authors acknowledge (e.g., lines 348-351) that limitations of many of the 55 constitutive publications do not allow a thorough stratification of pregnancy outcomes in Type 1 versus Type 2 diabetes, which would have been helpful, and should be addressed in future studies.

We have now added the following in response to the reviewer’s comments in the Discussion section under research recommendations,

‘*Further research is needed to better understand the associations between diabetic microvascular diseases and pregnancy outcomes, and whether this differs by type 1 or type 2 diabetes’*. *(page 23, line 494)*

39. Meaning of the phrase "at booking" (lines 304 and 427) might not be readily apparent to some readers whose first language is not British English.

We have replaced both instances with the phrase ‘*the time of the first antenatal consultation’* *(page 17, line 347 and page 23, line 485).*
